# Plasma sodium, extracellular fluid volume, and blood pressure in healthy men

**DOI:** 10.14814/phy2.15103

**Published:** 2021-12-18

**Authors:** Jacqueline J. J. O. N. van den Bosch, Niek R. Hessels, Folkert W. Visser, Jan A. Krikken, Stephan J. L. Bakker, Ineke J. Riphagen, Gerjan J. Navis

**Affiliations:** ^1^ Department of Internal Medicine Division of Nephrology University of Groningen University Medical Center Groningen Groningen The Netherlands; ^2^ Department of Internal Medicine Division of Nephrology Zorggroep Twente Almelo The Netherlands; ^3^ Department of Internal Medicine Division of Cardiology University of Groningen University Medical Center Groningen Groningen The Netherlands; ^4^ Department of Laboratory Medicine University of Groningen University Medical Center Groningen Groningen The Netherlands; ^5^ Present address: Department of Ophthalmology University of Groningen University Medical Center Groningen Groningen The Netherlands

**Keywords:** blood pressure, extracellular fluid volume, plasma sodium

## Abstract

In the general population we recently reported a consistent association between plasma sodium and volume markers, suggesting that individuals with higher plasma sodium have higher extracellular fluid volume (ECFV). To test this hypothesis, we analyzed the association between plasma sodium and directly measured ECFV (iothalamate distribution volume) in healthy men. Second, we studied whether plasma sodium is associated with blood pressure. We analyzed data from 70 men (age 24 ± 7 years) at the end of two 7‐day periods on a low‐sodium diet (LS, 50 mmol Na/24 h) and a high‐sodium diet (HS, 200 mmol Na/24 h), respectively. The association of plasma sodium with blood pressure was assessed in the combined data of the different sodium intakes by linear mixed effects models. A positive univariable association between plasma sodium and ECFV was found during HS (*β* = 0.24, *p* = 0.042) and LS (*β* = 0.23, *p* = 0.058), respectively. Individual values of plasma sodium on LS and HS diet were strongly correlated (*β* = 0.68, *p* < 0.001), as were values for ECFV (*β* = 0.54, *p* < 0.001). In the combined data set plasma sodium level was significantly associated with ECFV (B [SE] = 0.10 [0.04], *p* = 0.02), and systolic blood pressure (SBP, B [SE] = 0.73 [0.26], *p* = 0.006), independent of ECFV. In conclusion, plasma sodium concentration is positively associated with ECFV on both LS and HS intake. Our data confirm and extend prior data on individual regulation of plasma sodium and suggest that this is associated with individuality of the regulation of ECFV. Finally, plasma sodium level is associated with SBP, independent of ECFV and diet.

## Graphical abstract

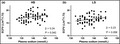

## 1 INTRODUCTION

Current concepts concerning the regulation of sodium‐ and volume homeostasis owe much to the Guyton hypothesis. According to this hypothesis, osmoregulation and volume regulation act in concert to control water and volume balance and, secondarily, blood pressure (Guyton, 1989). Briefly, increased sodium intake is believed to induce a subtle rise in plasma sodium, stimulating induction of thirst by the release of arginine vasopressin (AVP, also known as antidiuretic hormone (ADH)) and, subsequently, fluid transfer from intracellular to the extracellular space. The release of AVP, moreover, leads to renal water retention and hence excretion of concentrated urine (Bourque, 2008). The rate of renal sodium excretion increases gradually to the level where intake matches excretion again and steady state is restored, with a slightly higher extracellular fluid volume (ECFV). As a result, healthy humans are likely to tolerate a wide range of sodium intakes without clinically relevant changes in plasma sodium, volume status, or blood pressure (Guyton & Hall, 2006; Kumar & Clark, 2009; Roos et al., 1985).

This concept has been very influential over decades, and continues to raise interest as well as controversy (Böger et al., 2017; Kurtz et al., 2018; Machnik et al., 2009; Tian et al., 2009; Wilmot et al., 2012; Zerbe et al., 1991; Zhang et al., 2014). Due to methodological hurdles, the empirical support is partly derived from animal studies and computational modeling studies. Still, in recent decades, major advances in both computer software and in vivo techniques have improved abilities to test and recreate both existing and new hypotheses. For example, Kurtz et al. recently found that two computational derivatives of Guyton's 1972 model could not consistently predict changes in sodium balance and hemodynamic responses in normal subjects (Kurtz et al., 2018). Moreover, MacHnik et al. (2009) show that the interstitium can have tonicity that differs from plasma and suggest that a macrophage‐dependent pathway promotes sodium storage in the intersitium under high salt conditions. Taken together, this implicates that the regulation of intra‐ and extracellulair fluid is more complex than would be assumed according to the model of pressure natriuresis. Indeed, inhibition of this macrophage‐dependent pathway leads to increased sodium sensitivity of blood pressure. Lastly, recent studies describe the presence of genetic differences in osmoregulation and sodium handling, suggesting a certain individuality of plasma sodium regulation (Böger et al., 2017; Tian et al., 2009; Wilmot et al., 2012; Zerbe et al., 1991; Zhang et al., 2014). The aforementioned lines of evidence all illustrate the relevance of additional empirical data on the interrelationships of volume homeostasis and osmoregulation.

A key element in these interrelationships is the association between plasma sodium and ECFV. Based on the mathematical model by Guyton et al, one would not expect an alteration of plasma sodium in response to increased sodium intake after steady state has been restored, due to the expansion of the extracellular volume (Guyton, 1989). Yet, in a recent cross‐sectional study in the general population, we found an association between volume markers (NT‐proBNP and aldosterone, respectively) and plasma sodium, suggesting a weak, but consistent positive association between higher plasma sodium and a higher ECFV (Hessels et al., 2019). Unfortunately, direct data on ECFV were not available in the PREVEND cohort, as accurate ECVF measurements are notoriously challenging in humans, especially for large scale studies. As regulation of ECFV is also assumed to be involved in the regulation of blood pressure (Guyton & Hall, 2006), it would also be interesting to investigate whether plasma sodium is associated with blood pressure, and whether ECFV and/or sodium intake are involved in such an association.

Therefore, in the current study, we tested the hypothesis that plasma sodium is positively associated with ECFV in a small‐scale study in healthy subjects, with assessment of ECFV by a validated method, that is, iothalamate distribution volume (Amici et al., 1996; Visser et al., 2008). To this purpose, we studied whether plasma sodium is associated with ECFV in healthy subjects in balance on two different levels of sodium intake within the normal range, in a crossover design. Then, we studied the individual consistency of both plasma sodium and ECFV between two different levels of sodium intake. Lastly, we tested whether plasma sodium is associated with blood pressure and whether ECFV and/or sodium intake are involved in such an association.

## 2 METHODS

### 2.1 Study population

The present study is a post‐hoc analysis from a prior study of which the design and details have been published previously (Krikken et al., 2007; Visser et al., 2008, 2009). In short, healthy male volunteers followed a low‐sodium diet (LS) of 50 mmol Na/24 h and a high‐sodium (HS) diet of 200 mmol Na/24 h for 7 days in randomized order, interchanged by washout periods of at least 3 weeks. Potassium intake was standardized at 80 mmol per 24 h. Subjects continued usual food habits and normal activities during the entire study period. Compliance was tested by measurement of 24 h urinary sodium excretion on day 4 and 6. All subjects with available data on ECFV (*n* = 70/93) were included in the present study. The study was approved by the local medical ethics committee, in accordance with the Declaration of Helsinki Principles. All participants gave written informed consent.

### 2.2 Data collection and measurements

Careful assessment of steady state was performed by evaluating dietary compliance, body weight, and sodium balance by a 24‐h urine collection at day 4 and 6 of the study week. At day 7, subjects reported at 8 a.m. in the hospital while being abstained from food and alcohol. After a 2‐h run‐in period to achieve steady state of the continuously infused tracers, glomerular filtration rate (GFR), and effective renal plasma flow was measured by the urinary clearance of ^125^I‐iothalamate and ^131^I‐hippuran, respectively. The GFR was measured by urinary clearance of ^125^I‐iothalamate and corrected for voiding errors using the ratio of plasma to urinary clearance of ^131^I‐hippuran. ECFV was measured as the distribution volume of ^125^I‐iothalamate during steady state. The distribution volume of ^125^I‐iothalamate was calculated from the plasma level divided by the total amount in body, which equals the amount infused minus the amount excreted (Amici et al., 1996). The measured GFR, ERPF, and ECFV had a day to day variation of 2.5, 5, and 9.2%, respectively. At 11 a.m., blood was withdrawn for determination of plasma renin activity (PRA) and aldosterone concentration. Blood pressure was measured with an automatic device (Dinamap) every 3 for 15 min. BSA was calculated according to: 0.007184 × height (cm)^0.725^ × body weight (kg)^0.425^ (Du Bois, 1916). ECFV was indexed to 1.73 × BSA. Urinary concentrations of sodium and potassium and plasma concentrations of sodium were measured using an automated multi‐analyzer (Modular, Roche Diagnostiscs). Electrolyte free water clearance (EFWC) was calculated through the following formula: ECFW = V[1 − (*U*_na_ + *U*_K_)/[Na^+^]_p_], where *V* is the total volume, which was divided by 1440 in order to report ECFW in ml/min (Goldberg, 1981). Plasma renin activity was measured in terms of angiotensin I generation using a radioimmunoassay. Aldosterone concentration was determined with a commercially available radioimmunoassay kit.

### 2.3 Statistical analysis

Data collected at day 7 of both the LS and HS diet were included for each individual. Statistical analyses were performed using SPSS version 22.0 for Windows (IBM Corporation). Results are presented as mean ± standard deviation (SD) for variables with a normal distribution or as median [interquartile range] for variables with a skewed distribution. Nominal data were presented as number of participants with percentage. A two‐sided *p* < 0.05 was considered to indicate statistical significance.

Physiologic data of the participants during HS and LS are presented separately. *p*‐values for differences between parameters after the HS and LS diet were investigated using the paired *T*‐test for normally distributed data or the Wilcoxon signed‐rank test for nonparametric data.

To assess the association between plasma sodium and ECFV on LS and HS intake, we used univariate linear regression analysis on the data on LS and HS, respectively. To study the individual consistency of both variables during LS and HS, we performed intraindividual correlations of plasma sodium and ECFV, respectively. To assess the association of plasma sodium with blood pressure and ECFV we used the combined data on LS and HS, using linear mixed effects models for repeated measurements, to account for the change in diet and the repeated measures. We used the variance components covariance structure with “diet” and “plasma sodium” as fixed effects and “subject” as random effect, to assess the associations of plasma sodium level with levels of ECFV, systolic blood pressure (SBP), and pulse pressure (PP).

## 3 RESULTS

A total of 70 normotensive men with a mean age of 24 ± 7 years were included. Physiological data of the participants are presented in Table 1. Mean urinary sodium excretion was 38 ± 26 mmol/24 h during LS and 230 ± 67 mmol/24 h during HS (*p* < 0.001), indicating good dietary compliance. During LS, plasma sodium concentration, body weight, BMI, SBP, MAP, PP, ECFV, GFR, and ERPF were all significantly lower than during HS diet, while PRA, EFWC, and aldosterone were significantly higher during LS than during HS diet (*p* < 0.001; Table 1). No significant differences were found between HS and LS for diastolic blood pressure and urinary volume.

**TABLE 1:** Physiologic data after high‐ and low‐sodium diet (*N* = 70)

	High‐sodium diet	Low‐sodium diet	*p*‐value
Plasma sodium (mmol/L)	140 ± 3	138 ± 3	0.001
Body weight (kg)	80.6 ± 10.8	79.2 ± 10.5	<0.001
BMI (kg/m^2^)	23.4 ± 2.6	23.0 ± 2.6	<0.001
SBP (mmHg)	123 ± 10	120 ± 10	0.007
DBP (mmHg)	70 ± 7	69 ± 7	0.09
MAP (mmHg)	88 ± 7	86 ± 7	0.02
PP (mmHg)	53 ± 9	52 ± 9	0.04
BSA (cm*kg)	2.04 ± 0.15	2.03 ± 0.15	<0.001
ERPF (ml/min)	592 ± 96	559 ± 89	<0.001
GFR (ml/min)	138 ± 18	128 ± 18	<0.001
ECFV (L/1.73 m^2^)	17.4 ± 1.66	16.5 ± 1.54	<0.001
PRA (ng angL/ml/h)	2.10 (1.40–3.10)	5.74 (4.19–7.80)	<0.001
Aldosterone (ng/L)	39 (24–57)	134 (80–178)	<0.001
Urinary volume (ml)	1722 ± 674	1835 ± 743	0.2
Sodium excretion (mmol/24 h)	230 ± 67	38 ± 26	<0.001
Potassium excretion (mmol/24 h)	72 (61–93)	76 (55–100)	0.6
EFWC (ml/min)	−1.3 (−2.0; −0.6)	0.20 (−0.07; −0.40)	<0.001

Note

Values are in mean ± standard deviation or median (interquartile range) for continuous and categoric variables, respectively.

Abbreviations: BMI, body mass index; BSA, body surface area; DBP, diastolic blood pressure; ECFV, extracellular fluid volume; ERPF, effective renal plasma flow; GFR, glomerular filtration rate; MAP, mean arterial pressure; PP, pulse pressure; PRA, plasma renin activity; SBP, systolic blood pressure.

**FIGURE 1:**
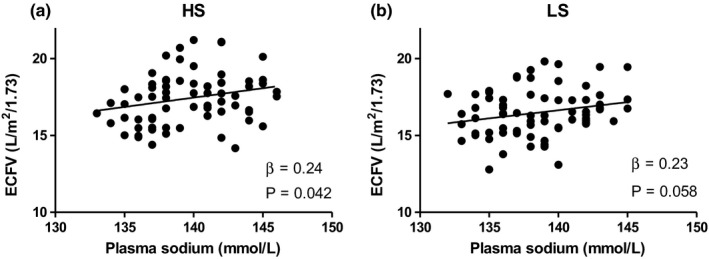
Scatter plots illustrating the association of plasma sodium concentration with ECFV (a, b) during HS and LS diet, respectively. ECFV, extracellular fluid volume; HS, high sodium; LS, low sodium; PP, pulse pressure; SBP, systolic blood pressure

**FIGURE 2:**
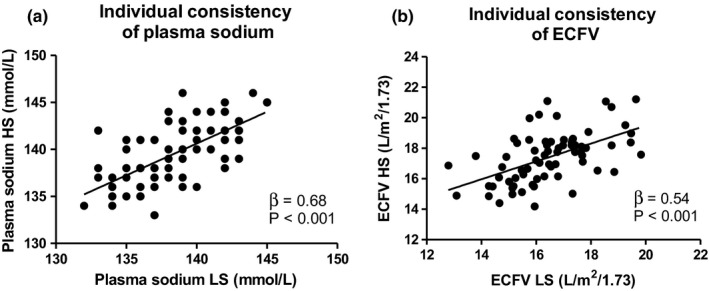
Individual consistency of plasma sodium (a) and ECFV (b) after a 7‐day high‐ and low‐ sodium diet. ECFV, extracellular fluid volume; HS, high sodium; LS, low sodium

Figure 1 shows the univariable associations of plasma sodium concentration with ECFV, during HS diet (*β* = 0.24, *p* = 0.042) and LS diet (*β* = 0.23, *p* = 0.058), respectively. Next, associations of plasma sodium during LS with plasma sodium during HS (*β* = 0.68, *p* < 0.001) and of ECFV during LS with ECFV during HS (*β* = 0.54, *p* < 0.001) are shown in Figure 2.

In the combined data set, mixed effect models for repeated measurements were used to adjust for diet and the strong individual consistency, plasma sodium level was significantly associated with ECFV (0.10 [0.04], *p* = 0.02). In addition, plasma sodium level was significantly associated with levels of SBP (B [SE] = 0.73 [0.26], *p* = 0.006) and PP (0.76 [0.20], *p* < 0.001; Table 2). No significant association was found between plasma sodium level and DBP. Plasma sodium level remained significantly associated with levels of SBP and PP after including ECFV in the model (Table 2).

**TABLE 2:** Associations of plasma sodium with SBP, PP, and ECFV using linear mixed effect models

	SBP		PP		ECFV	
	*Β* (SE)	*p*‐value	Β (SE)	*p*‐value	*Β* (SE)	*p*‐value
Model 1						
Plasma sodium (mmol/L)	0.73 (0.26)	0.006	0.76 (0.20)	<0.001	0.10 (0.04)	0.02
Diet (LS vs. HS)	−1.99 (1.02)	0.06	−0.65 (0.72)	0.4	−0.82 (0.19)	<0.001
Model 2						
Plasma sodium (mmol/L)	0.67 (0.26)	0.01	0.74 (0.21)	<0.001	–	–
Diet (LS vs. HS)	−1.49 (1.11)	0.2	−0.51 (0.80)	0.5	–	–
ECFV (L/1.73 m^2^)	0.60 (0.51)	0.2	0.17 (0.39)	0.7	–	–

Abbreviations: ECFV, extracellular fluid volume; HS, high sodium; LS, low sodium; PP, pulse pressure; SBP, systolic blood pressure.

## 4 DISCUSSION

The present study demonstrates that plasma sodium is positively associated with ECFV in healthy subjects in sodium balance on two different sodium intakes within the normal range. The association was present on either sodium intake, albeit of borderline significance during low sodium. Both plasma sodium and ECFV showed a strong intra‐individual correlation for values obtained during low‐ and high‐sodium intake. Analysis of the combined data on LS and HS, confirmed the significant association of plasma sodium with ECFV, and showed that plasma sodium level was associated with levels of SBP and PP, independent of ECFV and of diet.

The subtle change in plasma sodium concentration between a LS and HS diet in healthy subjects found in this study has been described previously (Wardener et al., 2004). Also, the finding that ECFV was significantly higher during HS compared to a LS diet in this population was described by our group (Visser et al., 2009). Interestingly, in the current study plasma sodium was positively associated with ECFV during both HS and LS. Thus, apparently, osmoregulation does not fully restore plasma sodium to its original value after reaching the new steady state. This is in line with the notion that gain of a physiological feedback loop is usually not infinite and restoration of a baseline situation after a disturbance is rarely complete (Young et al., 1976, 1977). The current study was not designed to elucidate underlying mechanisms, and unfortunately data on AVP or copeptin were not available. Nevertheless, several inferences can be made. We found that EFWC was significantly different during HS en LS, with a negative EFWC during HS. This might implicate that HS is driving water retention in order to lower the elevated plasma sodium, whereas the opposite was observed for these individuals during LS diet (Batlle et al., 2008). Thus, our study adds to the empirical data that hypo‐ and hypertonic diets induce subtle changes in plasma sodium, at least on the short term, consistent with the literature (Wardener et al., 2004). From a theoretical perspective, several possible mechanisms could be involved. For example, extrarenal pathways, such as extrarenal sodium storage (MacHnik et al., 2009) or individual differences in the complex hypothalamic–pituitary–adrenal (HPA) could play a role. Moreover, from pathophysiological studies it is known that humans are able to reset their osmostat under high salt or low salt conditions (Feder et al., 2019). This could possibly result from differences in arginine vasopressin (AVP) sensitivity among these individuals (Ettema et al., 2018). Our data might imply that also under physiological circumstances individuals are able to, at least to some extent, reset osmoregulation during LS or HS diets. Further studies would be needed to substantiate this assumption.

As mentioned previously, recent data support individuality of plasma sodium levels, and the presence of a heritable component for plasma sodium (Wilmot et al., 2012; Zhang et al., 2014). Whether the individuality of plasma sodium is affected by sodium intake is so far unknown. Our data confirm the individuality of plasma sodium and show that it is maintained over a considerable change in sodium intake within the normal range. Interestingly, we also observed a strong within‐individual correlation for ECFV on LS and HS intake, strongly suggesting that, in healthy subjects, regulation of ECFV is also an individual characteristic. In the combined data set, after accounting for diet and repeated measurements, plasma sodium was still significantly associated with ECFV. Taken together, this suggests the presence of a physiological feedback loop between the individually set plasma sodium and volume regulation. Thus, our data are in line with prior data on the individuality of plasma sodium and add that the individuality is maintained despite a significant effect of a change in sodium intake on average plasma sodium. Moreover, our data fuel the hypothesis that individuality of plasma sodium, based on genetic and environmental (sodium intake) drivers, is a major factor underlying individuality of ECFV in healthy subjects.

Regulation of ECFV is assumed to be involved in regulation of blood pressure as well (Guyton & Hall, 2006), and its derangement is assumed to be involved in volume‐dependent hypertension. Considering the association between higher plasma sodium and higher ECFV, we therefore investigated whether plasma sodium was associated with blood pressure, and whether ECFV was involved in such an association. Whereas plasma sodium was significantly associated with SBP and PP, ECFV did not contribute signficantly to the model in these normotensive healthy volunteers. The association between plasma sodium and systolic blood pressure has been studied in population studies (Bulpitt et al., 1986; Lago et al., 2008; Rinner et al., 1989; Wannamethee et al., 1994), but none observed a significant association with SBP. In fact, one study even reported a weak inverse association with DBP. A potential explanation for the discrepancy with our results could be our homogeneous, healthy study population, studied under standardized conditions. Our results do, however, corroborate data by Suckling et al. (2012) which observed a stronger association of plasma sodium concentration with SBP compared to DBP in response to an increase in salt intake in normotensive volunteers.

The significant association of plasma sodium with the systolic component of blood pressure observed in the present study was independent of ECFV. This does not refute a role for ECFV in blood pressure regulation, but co‐linearity of plasma sodium and ECFV in our data set precludes further assumptions in this respect. The predominance of sodium over ECFV in the model is consistent with assumption that plasma sodium is the driving force in the relationship between plasma sodium and ECFV. Yet, caution is warranted in making this inference, as the predominance of sodium in the model could also be due to methodological factors such as a better signal‐to‐noise‐ratio for plasma sodium. Our findings suggest that SBP is associated with plasma sodium through as yet unknown pathways. Exploring these unknown pathways could possibly lead to new insights for future clinical practice.

Similar to our findings, previous studies have reported a positive association between PP and a high‐sodium diet (Buyck et al., 2009; Redelinghuys et al., 2010). PP is related to arterial stiffness and might be influenced by age and sex (Du Cailar et al., 2004; Safar, 1996). Interestingly, plasma sodium has been observed to cause stiffening of vascular smooth muscle cells in high physiologic concentrations, by reducing the release of the vasodilator nitric oxide (Oberleithner et al., 2007). Effects of high plasma sodium on the microvascular glycocalyx might be involved as well (Oberleithner & Wilhelmi, 2015). Thus, high plasma sodium may cause arterial stiffness leading to a rise in PP, favoring a rise in SBP. In our study, individual plasma sodium levels were positively associated with PP regardless of sodium intake. We hypothesize that the young men who took part in the current study have compensatory mechanisms to prevent a rise in blood pressure during high salt intake. As increased PP is commonly observed in patients over 50 years of age with essential hypertension, in patients with hypertension and patients with kidney failure who are treated with hemodialysis, and in patients with hypertension and atherosclerosis, it would be interesting to investigate whether changes in plasma sodium concentrations and changes in PP in response to changes in sodium intake are augmented in these populations (Safar, 1996).

We studied physiology in healthy men, and hence inference of clinical implications is not warranted. Our current data do provide mechanistic evidence in line with our prior observations in the PREVEND cohort (Hessels et al., 2019), suggesting that the association of plasma sodium with volume markers we observed in the general population is indeed driven by ECFV. The other way round, the consistency between the studies suggests that the current findings, obtained under controlled study conditions, are robust––at least to some extent––when generalized to the general population. Still, our data should not be extrapolated to conditions of volume‐dependent hypertension, such as in cardiorenal patients, where pathophysiological alterations in sodium handling as well as osmoregulation can occur.

Several limitations of our study should be considered. First, given the observational design of this study, no conclusions about underlying (causal) mechanisms can be drawn. Second, by the inherent limitations of well‐controlled dietary intake over prolonged periods, the duration of each study period was limited to 1 week. Whereas this is sufficient to restore steady state in terms of a urinary sodium excretion that matches intake again, other homeostatic adaptations can occur later on. Therefore, we cannot be certain that our results can be extrapolated to long‐term conditions. Moreover, our study design did not allow to account for the dynamics of sodium balance, that is, now known to include rhythmic excretory and retention patterns (Rakova et al., 2013). Furthermore, we studied a healthy group of relatively young men. As a result, our data cannot be extrapolated to populations with disease conditions, such as cardiorenal disease or other population groups such as females and elderly. Strengths of our study are the homogeneity of our data, the well‐controlled study conditions, as well as the measurement of ECFV by a well‐validated method.

In conclusion, in this study we found that plasma sodium is positively associated with ECFV in healthy subjects in sodium balance on two different sodium intakes within the normal range. This suggests that plasma sodium is involved in the regulation of ECFV under normal conditions. A clear individuality of plasma sodium and ECFV was apparent. In addition, we found that plasma sodium level was associated with systolic SBP and PP, independent of ECFV and of diet. It would be interesting for future studies to investigate the link between plasma sodium and blood pressure. Exploration of this yet unknown pathway could possibly lead to new insights in (patho‐)physiology, and, eventually future clinical practice.

## DISCLOSURES

The authors have no competing interests.

## AUTHOR CONTRIBUTION

JJONVdB (shared 1st author): data analysis and interpretation, writing manuscript, and final approval. NRH (shared 1st author): data analysis and interpretation, writing manuscript, and final approval. FWV: design, data collection, manuscript revision, and final approval. JAK: design, data collection, manuscript revision, and final approval. SJLB: data interpretation, manuscript revision, and final approval. IJR: data analysis and interpretation, manuscript revision, and final approval. GJN: design, data analysis and interpretation, manuscript revision, and final approval.
